# Integrating mental health into fracture liaison services: an attribution-first policy approach to depression, concerns about falling and pathway loss

**DOI:** 10.3389/fpubh.2026.1989494

**Published:** 2026-10-26

**Authors:** Fujin Liao, Fengtao Zhou, Xin Li, Zhengchun Wang, Qiusheng Wang

**Affiliations:** 1 Shenzhen Pingle Orthopedic Hospital (Shenzhen Pingshan District Hospital of Traditional Chinese Medicine), Shenzhen, Guangdong, China; 2 Division of Arts, Shenzhen University, Shenzhen, Guangdong, China

**Keywords:** concerns about falling, depression, fracture liaison service, implementation science, patient engagement, public mental health, quality indicators, secondary fracture prevention

## Abstract

Fracture liaison services (FLSs) improve delivery of secondary fracture prevention, yet eligible patients are still lost between identification, assessment, treatment recommendation, initiation, rehabilitation and follow-up. This Policy and Practice Review asks whether depression and concerns about falling should inform FLS policy, and how they could be addressed without displacing core fracture-prevention work. We synthesized structured PubMed searches with targeted appraisal of the International Osteoporosis Foundation Capture the Fracture architecture, cross-national FLS studies, clinical guidelines, audit documents and implementation-science frameworks. Evidence, derived mainly from hip-fracture populations, links depressive morbidity and mental-disorder indicators to poorer recovery or lower treatment initiation, and links concerns about falling to activity restriction and functional outcomes. These findings are prognostic rather than causal: pain, frailty, cognition, environment and service organization remain incompletely controlled. Intervention studies can improve mood or falls-related confidence without consistently improving objectively measured behavior, and no trial identified in this review used completion of an FLS engagement node as its primary outcome. Existing international and national frameworks make core FLS processes measurable, but they do not provide a consistent, auditable linkage between a non-completed node, the source of the attributed reason, the response delivered and the subsequent outcome. The England and Wales Fracture Liaison Service Database (FLS-DB) is used as a worked policy case: it records selected states such as non-attendance, patient decline and non-contactability, but its node-specific denominators and distributed fields cannot reconstruct the whole pathway. We compare four options: the status quo; cross-node reason attribution; targeted case finding linked to a named response; and a universal stepped pathway. Current evidence supports a staged sequence—attribution first, targeted measurement only where response capacity exists, and multicentre evaluation before universal standards. Evaluation should protect informed refusal, report reach and equity, preserve existing FLS performance and use prespecified stopping rules. Mental-health integration should therefore be treated as a testable quality-improvement strategy, not an established standard of care.

## 1 Introduction

Fragility fracture is both a sentinel clinical event and a time-limited opportunity for secondary prevention. It is associated with pain, functional decline, loss of independence and excess mortality, while the risk of another fracture is highest soon after the index event (1, 2). A public-health response must therefore do more than treat the acute injury: it must identify eligible patients rapidly and convert the fracture into sustained prevention.

Fracture liaison services (FLSs) were developed to organize this response through systematic identification, fracture-risk and bone-health assessment, treatment recommendation, falls or rehabilitation intervention and follow-up (3). Controlled studies and meta-analyses show that FLSs improve bone mineral density testing and anti-osteoporosis treatment initiation, and are associated in some settings with better persistence and fewer subsequent fractures (4–6). The evidence supports the model, but leaves a different policy question unresolved: why an eligible patient does not always complete the next indicated step even when an FLS exists.

In this review, engagement is not treated as a synonym for medication adherence. It is a sequence of node-specific events: attending assessment, understanding risk, participating in a treatment decision, accepting or declining treatment, initiating medication, entering rehabilitation, completing follow-up and sustaining longer-term management. These events have different determinants and different responsible actors. A considered refusal after an accessible, balanced discussion is a legitimate outcome; failure to contact a patient, provide an accessible appointment or coordinate follow-up is a service failure. Combining both under 'non-adherence' prevents a service from locating, and therefore correcting, the relevant loss.

Depression and concerns about falling merit examination because each is common after fracture, potentially modifiable and plausibly relevant to a specific node. Depressive morbidity has been associated with poorer recovery and, in administrative cohorts, with lower initiation of osteoporosis treatment; concerns about falling have been associated with activity restriction and functional recovery (7–10). The evidence is dominated by hip-fracture populations and uses heterogeneous measures. Pain, cognition, health beliefs, social support, communication and access may be equally or more important influences on pathway participation. In this review, depression and concerns about falling serve as two clinically salient probes through which to examine how FLS policy can recognize and respond to psychosocial influences at specific care transitions.

The policy gap is narrower than an absence of psychosocial awareness. International and national frameworks specify core FLS processes, and the England and Wales audit system records selected states at several nodes. However, existing indicators primarily establish whether a service step occurred; they do not consistently link every non-completed transition to a reason, the source of that attribution, a named response and the subsequent outcome. Follow-up and persistence indicators are also conditioned on prior treatment recommendation, limiting reconstruction of loss across the whole eligible pathway. The resulting policy problem is insufficient explanatory resolution and accountability across successive care transitions.

This Policy and Practice Review evaluates four options: maintaining the status quo; adding cross-node reason attribution; introducing targeted case finding linked to a predefined response; and adopting a universal stepped pathway. It synthesizes empirical evidence on depression and concerns about falling with international standards, clinical guidance, audit materials and implementation frameworks. The central proposition is staged: make pathway loss attributable first, add targeted measurement only where a response can be delivered, and test the combined pathway across multiple FLSs before considering universal indicators. By locating psychological assessment within a node-specific accountability framework, the review translates a broad call for mental-health integration into a proportionate, auditable and testable policy strategy. Its contribution is to distinguish informed choice from remediable barriers, connect assessment to a named response and define how added activity can be evaluated while preserving core secondary fracture-prevention functions.

## 2 Review approach

### 2.1 Review design and policy question

This article was developed as a critical narrative Policy and Practice Review rather than a systematic review or meta-analysis. It brings together three bodies of literature that have generally been examined separately: the organization and measurement of FLSs; depression and concerns about falling after fragility fracture; and implementation approaches for translating clinical recommendations into sustained patient participation. The principal unit of analysis was the FLS care-pathway node rather than a diagnosis, psychological construct or intervention considered in isolation. Evidence was considered relevant when it informed at least one of four questions: where non-completion occurs; whether depression or concerns about falling are associated with a subsequent recovery or engagement outcome; whether an intervention modifies the proposed psychological mechanism or related behavior; and whether an FLS could identify and respond to that mechanism without weakening its core fracture-prevention functions.

### 2.2 Information sources and search strategy

Four structured PubMed searches were conducted on 31 August 2026 across four domains: FLS policy and quality measurement; FLS implementation and engagement; depression and post-fracture osteoporosis-treatment engagement; and concerns about falling and post-fracture rehabilitation or participation. The searches retrieved 240, 397, 121 and 152 records, respectively. The 910 records across searches contained 164 cross-search duplicates, leaving 746 unique PubMed records. Exact strategies are provided in Supplementary Table S1A and Supplementary Dataset S1. Source-selection accounting was reconstructed from the preserved search outputs, the source-verification ledgers and the final reference set, and was verified on 15 September 2026. Of the 746 unique PubMed records, 20 reports were assessed in full text and 19 were retained; 726 title/abstract records were not retained. Targeted policy, guideline and audit searches and backward citation checking contributed 21 additional full-text reports to the original review, of which 19 were retained. Three measurement-validation reports were added during revision to operationalise case finding (11–13). Across routes, 44 full-text reports were assessed, three were excluded for the report-specific reasons stated in Supplementary Table S2B, and 41 sources were included in the revised synthesis. Supplementary Figure S1 presents the reconstructed source-selection flow, Supplementary Table S2 lists each full-text decision and exclusion reason, Supplementary Table S3 provides the evidence map, and Supplementary Tables S4, S5 provide the taxonomy codebook and case-finding specification. Contemporaneous item-level title/abstract exclusion reasons were not retained; the selection account is therefore reported as a retrospective reconstruction rather than prospective screening data.

### 2.3 Evidence selection and analytic framework

Priority was given to international and national standards, systematic and scoping reviews, controlled evaluations, population-based cohorts, qualitative studies reporting patient or clinician explanations for non-completion, and intervention studies reporting psychological, behavioral, functional or service outcomes. Evidence labels were applied consistently. Direct evidence was generated within an FLS or by an FLS-specific framework or audit and addressed an identifiable pathway process. Indirect evidence was generated in post-fracture or osteoporosis populations outside a defined FLS and could support plausibility or measurement, but not an FLS-specific causal claim. Contextual evidence came from adjacent older-person guidance or implementation and measurement frameworks. Author-derived inference was not counted as an evidence source; it comprised the proposed cross-node taxonomy, causal hypotheses, policy-option appraisal and staged implementation sequence, all of which are described as proposed and unvalidated. The evidence map assigns every included source a primary domain, source type, directness label and maximum permissible claim.

The 41 included sources were assigned to one mutually exclusive primary evidence domain: FLS model, effectiveness and implementation (*n* = 14); policy, guideline, audit and integrated care (*n* = 7); depression or mental disorder after fracture (*n* = 5); concerns about falling and rehabilitation (*n* = 6); treatment beliefs, non-initiation and shared decisions (*n* = 4); implementation and evaluation frameworks (*n* = 2); and measurement validation added during revision (*n* = 3). These counts describe sources used in the synthesis, not effect-estimate pools.

For each policy option, the synthesis considered directness and consistency of evidence, expected benefit, implementation burden, response capacity, patient autonomy, equity, opportunity cost and potential effects on existing FLS performance. Recommendations extending the FLS pathway were required to identify the relevant care node, responsible actor, action triggered, information recorded and condition under which the proposed addition should be modified or stopped.

### 2.4 Limits of the review approach

The synthesis followed an explicit hierarchy of inference. Associations between a psychological measure and a later outcome were treated as prognostic or correlational signals unless temporality, adjustment and design supported a stronger interpretation. Administrative mental-disorder diagnoses, antidepressant dispensing and symptom-scale elevations were treated as different constructs. Concerns about falling were distinguished from objectively assessed falls risk, and improvements in symptoms or confidence were not assumed to imply improvements in medication initiation, rehabilitation attendance, physical activity or refracture.

Because this was a critical narrative review, study selection and extraction were not performed independently by two reviewers, no *de novo* study-level risk-of-bias assessment was undertaken and no pooled effect estimate was calculated. The flow diagram and evidence map improve auditability but do not convert the work into a systematic review. Database searching was restricted to PubMed; the initial record count from targeted website searches was not preserved; and title/abstract exclusion reasons were reconstructed retrospectively from the retained source set. The absence of a study from this synthesis should not be interpreted as evidence that no such study exists. The revised synthesis includes 41 sources spanning empirical studies, evidence syntheses, policy documents, clinical guidance, implementation frameworks and measurement validation.

## 3 Assessment of current FLS policy and quality architecture

### 3.1 International standards define the core service, but not the causes of pathway loss

International FLS policy is anchored by complementary measurement systems. The IOF Capture the Fracture Best Practice Framework assesses services at organizational level through 13 standards covering identification, assessment, falls prevention, treatment initiation, communication, long-term management and database capability (14). The patient-level indicator set developed by the IOF Capture the Fracture Working Group, the Fragility Fracture Network and the National Osteoporosis Foundation complements this framework with 11 key performance indicators (KPIs) adapted from the UK-based Fracture Liaison Service Database (FLS-DB) audit (15). Together, these instruments establish a defensible international core: eligible patients should be identified, assessed promptly, considered for anti-osteoporosis treatment and falls intervention, and monitored after the initial recommendation.

The policy chain operates at several levels. IOF defines organizational scope and measurable patient-level processes; NOGG and NICE provide clinical guidance; the Royal Osteoporosis Society translates expectations into FLS service standards; and RCP/FFFAP operationalises selected processes through audit (2, 14–18). Clinical guidance, service standards, audit fields and performance indicators are not interchangeable policy instruments. A proposed mental-health addition must specify where it belongs.

This architecture is appropriately centered on services that FLS teams can deliver and measure. It makes visible whether fracture-risk assessment occurred, treatment was recommended, medication was initiated and follow-up was documented. It also separates stages sometimes collapsed into 'adherence'. Treatment recommendation, initiation and continued prescription are different events, with different responsible actors and potentially different causes of non-completion. This separation is an important strength and should be preserved in any mental-health-informed extension.

The limitation is explanatory rather than structural. Existing indicators record attainment of core care steps more consistently than they record why a patient did not progress between them. A falls-risk assessment does not establish whether concern about falling is restricting activity or rehabilitation. A documented treatment recommendation does not consistently distinguish informed refusal, unresolved treatment concerns, depressive morbidity, cognitive or communication difficulty, inability to access prescribing or failure of clinical follow-up. These states have different policy implications and should not be represented by a single category of patient non-adherence.

The KPI developers explicitly deferred patient-reported outcome measures because of licensing, administration, technical support and data-processing requirements (15). They did not conclude that patient-reported outcomes lacked value; rather, measurement should be introduced only when services can collect and use the information reliably. Adding a questionnaire is not a neutral extension of audit. It creates obligations to interpret the result, identify the responsible clinician, provide or coordinate a response and evaluate whether the added process improves care.

### 3.2 Cross-national evidence supports adaptation rather than uniform replication

The international transfer of FLS standards demonstrates both their broad applicability and the need for contextual adaptation. An Asia-Pacific consensus exercise concluded that the 13 IOF standards were generally applicable across the region while recommending clearer patient and family education and stronger public communication (19). A systematic review of Asia-Pacific services identified funding, staffing, interdepartmental coordination, primary-care participation and sustained follow-up as recurrent implementation needs (20). Variation in FLS performance therefore cannot be attributed solely to patient behavior; it also reflects how responsibilities, resources and transitions are organized.

Implementation studies reinforce this point. In Alberta, evaluation using the Reach, Effectiveness, Adoption, Implementation and Maintenance (RE-AIM) framework showed that an inpatient hip-fracture liaison service could improve osteoporosis-treatment initiation, while a linked qualitative study identified organizational readiness, resources, site selection and change management as important determinants (21, 22). In Australia, a Delphi study reached consensus on patient education, communication and the general practitioner-patient relationship during transition from FLS to primary care, but found continuing uncertainty about responsibility for adherence monitoring and long-term management (23). These studies locate engagement within a service system rather than treating it as a stable patient characteristic.

International benchmarking also illustrates the limits of applying an identically labeled KPI without examining the underlying service model. A Chinese hospital-based audit adapted FLS-DB indicators to an orthogeriatric inpatient pathway and reported strong inpatient assessment and treatment performance alongside marked post-discharge loss (24). The investigators distinguished the original dual-energy X-ray absorptiometry (DXA) indicator from a locally adapted DXA-or-quantitative computed tomography (QCT) measure. A common quality framework enables comparison, but identical labels may represent different clinical processes, denominators and opportunities for intervention.

Adjacent guidance offers relevant principles but does not close the FLS-specific gap. Falls guidance recommends multifactorial assessment and tailored intervention, while the World Health Organization Integrated Care for Older People (ICOPE) guidance includes mobility, psychological capacity, cognition and social support within person-centered assessment and care planning (16, 25, 26). These documents do not specify when an FLS should assess depression or concerns about falling, which engagement node the assessment is intended to protect, who should respond, or how success should enter FLS quality measurement.

Taken together, the international evidence supports a common core with locally specified implementation. It does not support adding a universal psychological screening target independently of workforce, referral capacity and the organization of follow-up.

Table 1 compares the contributions and unresolved limitations of the principal policy, quality-measurement and implementation sources considered in this review. Together, they support an internationally anchored FLS model while showing that no current framework provides a complete, auditable pathway linking psychological or organizational reasons for non-completion to a named service response.

**Table 1:** Policy, quality-measurement and implementation sources relevant to mental-health-informed fracture liaison service design.

Source, framework or evidence base	Primary level of application	Established contribution to FLS policy or practice	Implication for the proposed explanatory layer	Unresolved issue
IOF Capture the Fracture Best Practice Framework (14)	Service and organizational	Defines internationally recognized standards for the organization and delivery of core FLS functions.	Establishes the core functions that any additional assessment or response must preserve.	Does not specify FLS–specific case finding, response thresholds or accountable pathways for depressive symptoms or concerns about falling.
International patient–level FLS key performance indicators (15)	Patient and pathway measurement	Operationalises completion of specified patient–level FLS processes and outcomes.	Provides node–level anchors to which reason attribution and subsequent responses could be linked.	Does not consistently connect non–completion with its reason, information source, service response and subsequent outcome.
Asia–Pacific consensus statements and regional FLS evidence (19, 20)	Regional policy and implementation	Demonstrates that core FLS functions are transferable but require adaptation to local workforce, referral and care arrangements.	Shows that education, communication, staffing and coordination can condition pathway participation.	Provides no standardized cross–node approach to psychological case finding or response, and regional evidence remains heterogeneous.
Implementation studies from Canada and Australia (21–23)	Health–system and service implementation	Identify organizational readiness, professional roles, transfers of responsibility and continuity across care settings.	Frames pathway completion as co–produced by patients, professionals and service design rather than as patient adherence alone.	These studies were not designed to test an FLS mental–health pathway, and ownership of longer–term support remains variable.
Chinese hospital–based FLS KPI audit (24)	Hospital implementation and benchmarking	Demonstrates local adaptation of KPI–based measurement and identifies differences between acute and post–discharge performance.	Provides an international example in which early process delivery can coexist with later pathway attrition.	Does not establish whether depressive symptoms or concerns about falling explain transition non–completion.
England and Wales FLS–DB (RCP/FFFAP) (18, 27, 28)	National clinical audit in England and Wales	Benchmarks patient–level FLS processes at scale and records selected states of non–completion.	Provides an existing audit architecture in which limited, source–attributed reason fields could be evaluated.	Reason data remain partial across nodes and are not consistently linked to their source, service response or subsequent completion.
WHO Integrated Care for Older People guidance (26)	Global health–system guidance	Supports person–centered, integrated assessment linked to coordinated responses for declines in intrinsic capacity.	Provides an adjacent principle for assessing psychological or functional needs only when an actionable response is available.	Does not define FLS–specific thresholds, responsible actors, referral pathways or audit fields.
National falls and osteoporosis guidance (2, 16, 25)	National clinical guidance	Supports multifactorial falls assessment and evidence–based secondary fracture prevention.	Recognizes functional and psychological concerns that may affect rehabilitation and participation.	Does not define cross–node reason attribution or an accountable FLS–specific response to depression or concerns about falling.

Sources are grouped by their principal policy or implementation function and are not ranked as if they had equivalent evidentiary strength or normative authority. International frameworks, consensus statements, national clinical guidance, service standards, clinical audits and implementation studies serve different purposes. The columns describing implications and unresolved issues represent the authors' critical synthesis rather than positions formally endorsed by the issuing organizations. The England and Wales FLS–DB is used as a worked national–audit case and not as an international standard. FFFAP, Falls and Fragility Fracture Audit Programme; FLS, fracture liaison service; FLS–DB, Fracture Liaison Service Database; IOF, International Osteoporosis Foundation; KPI, key performance indicator; RCP, Royal College of Physicians; WHO, World Health Organization.

### 3.3 The England and Wales FLS-DB as a worked policy case

The England and Wales FLS-DB provides a useful worked case because it connects patient-level data, national audit and service benchmarking within a mature FLS system. It should not be treated as a universal model. Its value lies in showing how an operational audit represents the fracture-prevention pathway and where additional explanatory information could realistically be incorporated.

The 2026 annual report covers care delivered during 2024 to more than 83,500 patient records for people aged 50 years or older across 77 participating FLSs in England and Wales (18). Audit coverage was estimated at 63% of acute hospitals treating fragility fractures, and 42% of services achieved at least 80% data completeness across KPIs 2–11. Reported national results were 50% for non-spine identification, 38% for spine identification, 69% for bone-health assessment within 90 days, 41% for DXA within 90 days, 62% for falls assessment, 57% for treatment recommendation, 9% for strength and balance commencement, 32% for 12–16-week monitoring contact, 37% for treatment commenced or continued by first follow-up, and 30% for treatment recorded at 12 months. Three Northern Ireland services submitted data but were excluded from the published analysis because the audit was not commissioned there; these figures should therefore not be presented as a UK-wide estimate.

The percentages are not a single cohort funnel. Their denominators, eligibility rules and time windows differ: identification uses estimated caseloads; assessment and falls indicators use submitted records; DXA applies eligibility exclusions; and follow-up, initiation and 12-month indicators are conditioned on prior treatment recommendation or referral, with death, patient decline and non-contactability handled differently (18, 27). The audit can locate low-performing processes but cannot reconstruct the probability that the same eligible patient passes through every transition.

The system is not devoid of engagement or mental-wellbeing activity. The underlying dataset records selected states such as non-attendance, patient decline and non-contactability, while the RCP/FFFAP repository includes patient-engagement resources and the Stronger inside and out mental-wellbeing leaflet (18, 27, 28). The narrower limitation is that these states are distributed across node-specific fields and are not consistently linked to multiple contributing reasons, attribution source, named response and subsequent engagement outcome.

### 3.4 The policy gap is incomplete attribution and response

The central policy gap is not that FLS frameworks ignore patients, mental health or falls. It is that current performance data cannot consistently determine why an eligible patient did not move from one care node to the next or whether the service responded appropriately to the barrier identified.

This limitation is partly produced by indicator design. Follow-up, treatment-initiation and 12-month treatment indicators are conditioned on a prior treatment recommendation or referral and handle death, patient decline and non-contactability differently. A patient lost before recommendation does not enter later denominators; a patient recorded as declining may be excluded; and a non-contactable patient may remain in the 12-month denominator (27). These rules are defensible for the indicators concerned, but they cannot reconstruct loss across the whole eligible pathway or determine whether non-completion reflected an informed decision, an accessibility barrier, psychological difficulty or failure of service coordination.

Table 2 summarizes how these limitations operate across key FLS-DB pathway nodes and identifies the minimum prospective extensions required to test the proposed explanatory layer.

**Table 2:** What the England and Wales FLS–DB can and cannot explain at key pathway nodes.

Pathway node	Current measure and denominator	Existing attribution	What remains unexplained	Minimum extension to test
Identification	KPI 2/3 compare submitted records with estimated non–spine/spine caseloads.	Coverage and variation between services can be quantified.	No patient–level reason exists for a person never captured by the FLS.	Parallel local case–finding denominator with source, exclusions and unresolved cases.
Bone–health assessment	KPI 4: assessment within 90 days among submitted records.	Whether and when assessment occurred can be measured.	Non–completion is not linked consistently to patient, service or shared reasons and subsequent response.	Reason + attribution source + responsible owner + outcome at next contact.
DXA	KPI 5 applies eligibility exclusions and reports completion within 90 days.	The analysis plan can identify selected states, including did-not-attend.	The reason for non-attendance, accessibility response and later completion are not linked as a common audit chain.	Node-specific reason, source, adaptation offered and rescheduled outcome.
Falls and rehabilitation	KPI 6 records falls assessment; KPI 8 records commencement of strength and balance activity in an eligible subgroup.	Assessment/referral/commencement can be counted.	Objective falls risk does not reveal concern–related avoidance; commencement does not establish attendance or completion.	Separate activity–limiting concern from objective risk and record participation outcome.
Treatment recommendation and initiation	KPI 7 uses submitted records; KPI 10 is conditioned on recommendation/referral and excludes recorded death/decline.	Recommendation, initiation and selected decline states are distinguishable.	Conditioning obscures upstream loss; a decline may be informed, inaccessible or barrier–related, and its source is not consistently linked.	Retain official KPI; add all–eligible transition denominator and source–attributed reason.
Follow–up and 12–month treatment	KPI 9/11 use treatment–recommended or referred populations with node–specific exclusions; KPI 11 includes non–contactable patients.	Contact and persistence can be measured in the defined subgroup.	The system cannot reconstruct cross–node loss or show whether a named response changed later engagement.	Contact outcome, reason, response owner and reassessed engagement node.

Current measures and denominator rules are summarized from the England and Wales FLS–DB annual report and its 2026 analysis plan (18, 27). KPI percentages do not form a sequential patient funnel because eligibility criteria, exclusions and denominators differ between indicators. The proposed minimum extensions are an author–developed implementation dataset for prospective testing, not current FLS–DB requirements and not a retrospective reinterpretation of official audit performance. A non–completed step should not be equated automatically with non–adherence; informed refusal remains a legitimate outcome. FLS, fracture liaison service; FLS–DB, Fracture Liaison Service Database; KPI, key performance indicator.

A proportionate extension would begin with cross-node reason attribution rather than universal psychological screening. The author-developed taxonomy is a proposed data structure, not a validated classification. At each non-completed node, services should record one primary reason and any secondary contributing reasons; keep patient, caregiver, clinician and administrative accounts source-labeled; retain disagreement rather than forcing consensus; and allow “unknown”. Operational categories are unsuccessful contact; informed refusal; unresolved uncertainty or treatment concern; clinical ineligibility or deferral; cognitive or communication support need; patient-reported psychological barrier; practical or social access barrier; transfer or service failure; and unknown or mixed. Psychological attribution requires patient/caregiver report or documented clinical assessment that links the factor to the named node; a questionnaire score or missed appointment alone is insufficient.

Accordingly, this review does not propose replacing existing IOF standards or expanding FLS into a general mental-health service. The policy question is whether limited additions to attribution, targeted case finding and response pathways could explain and reduce otherwise preventable loss while preserving core fracture-prevention performance.

Figure 1 maps the existing FLS pathway against an attribution-first explanatory layer and the proposed staged policy response.

**Figure 1:**
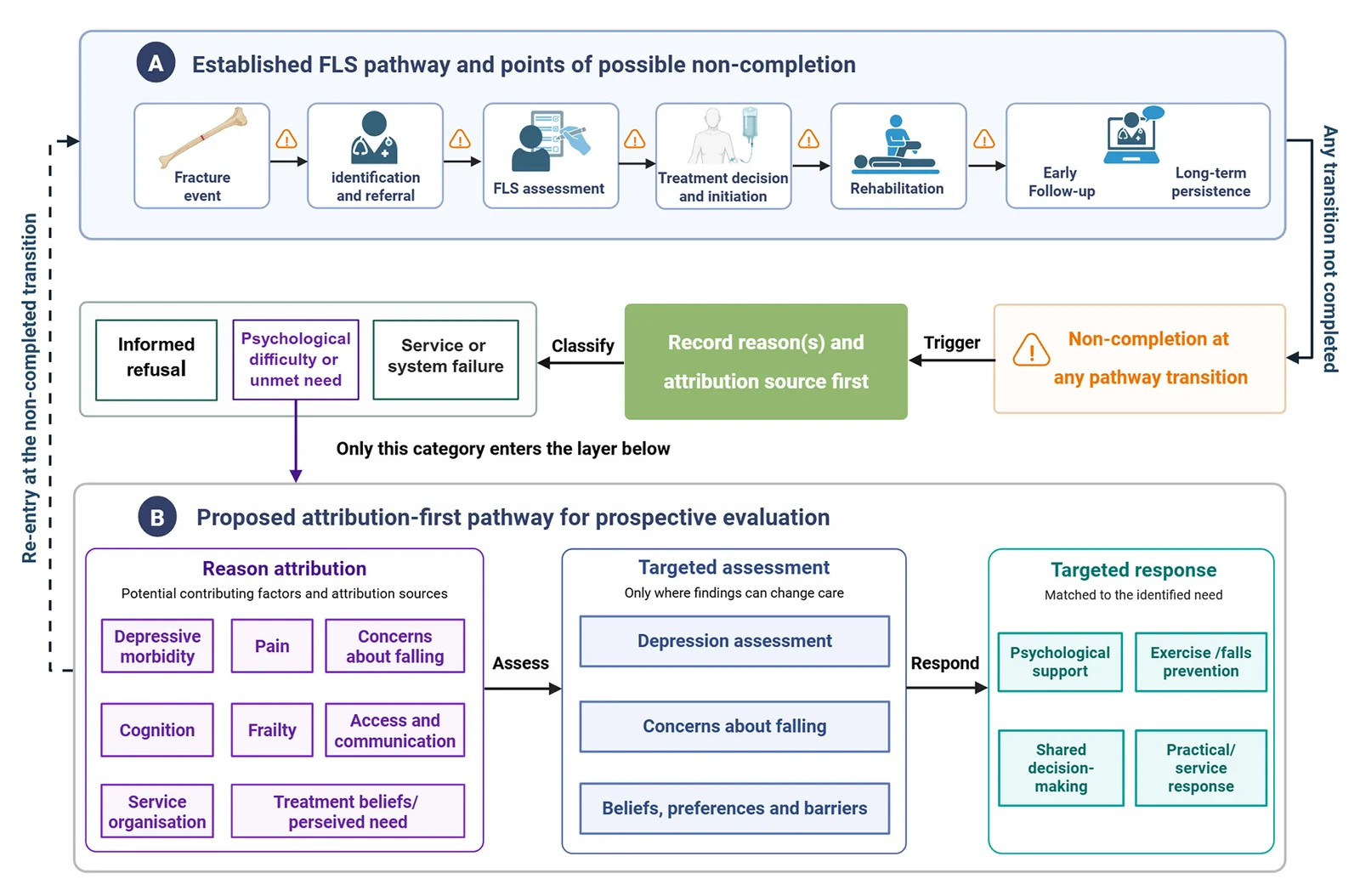
Existing fracture liaison service pathway and a proposed attribution-first response layer. **(A)** The established fracture liaison service (FLS) pathway extends from the fracture event through identification and referral, FLS assessment, treatment decision and initiation, rehabilitation, early follow-up and long-term persistence. Orange warning symbols indicate transitions at which pathway completion may be lost. When a transition is not completed, the proposed approach first records the contributing reason or reasons and their attribution source. Informed refusal following an accessible and balanced discussion should be recorded and respected; psychological difficulty or unmet need may warrant further assessment; and service or system failure should prompt service-level corrective action. **(B)** For potentially remediable non-completion, the proposed and currently unvalidated layer links reason attribution to targeted assessment and a response matched to the identified need. Depression and concerns about falling are treated as potential contributors or markers rather than presumed causes. Targeted assessment should be undertaken only when the findings could change care and an appropriate response is available; it does not represent universal psychological screening. Responses may include psychological support, exercise or falls-prevention intervention, shared decision-making, or a practical or service-level response. The feedback arrow denotes reassessment and re-entry at the transition that was not completed. Blue represents established FLS processes, orange indicates possible non-completion, purple represents reason attribution, blue-gray represents targeted assessment, and teal represents targeted responses. The proposed layer is intended for prospective evaluation and should not be interpreted as an established standard of care. FLS, fracture liaison service. Green denotes the proposed reason-recording step BioRender.com.

## 4 Evidence informing policy options

### 4.1 Depression: a plausible risk marker with limited FLS-specific evidence

Depressive morbidity after fragility fracture comprises several clinically different states: a pre-existing depressive disorder, symptoms emerging during acute admission, and symptoms persisting into rehabilitation and secondary prevention. These states should not be treated as interchangeable. Administrative diagnoses, antidepressant dispensing and elevations on symptom scales identify overlapping but different populations, and they may relate to different points in the FLS pathway.

The burden is clinically relevant but difficult to quantify across the full FLS population. A meta-analysis of 27 studies involving 11,958 older people with hip fracture estimated a pooled depression prevalence of 23%, although estimates varied by setting, measurement method and follow-up period (29). A broader scoping review identified depression as the most frequently studied psychological factor after hip fracture, examined in 49 of 55 included articles (7). However, the predominance of surgically treated hip-fracture cohorts limits generalization to patients with vertebral, wrist, humeral or pelvic fractures who may enter FLS through different pathways.

Evidence linking depression to recovery is suggestive rather than conclusive. In the scoping review, depressive symptoms were frequently associated with poorer physical function, mortality or other recovery outcomes, but findings varied across outcomes and instruments (7). A systematic review of factors predicting depression after hip-fracture surgery identified 37 candidate prognostic factors in 12 studies, with conflicting results; all 12 studies were at high risk of bias in at least one domain, and no study was at low risk across all domains (30). These reviews support depression as a marker of vulnerability; they do not establish a sufficiently stable causal pathway on which an FLS policy could yet be based.

The evidence most directly relevant to FLS policy concerns treatment initiation. In a Danish population-based cohort of 57,887 patients aged 65 years or older with hip fracture, the one-year cumulative incidence of anti-osteoporosis medication initiation was 14.8% among patients with a recorded mood disorder and 20.8% among those with no recorded mental disorder (9). Lower initiation was also observed for organic, schizophrenic, substance-use and anxiety disorders, indicating that the treatment gap was not specific to depression. A US Medicare analysis similarly found that antidepressant use was associated with lower post-fracture osteoporosis-treatment initiation in both women and men (10).

These findings map to an established FLS node, but they do not demonstrate that depression caused non-initiation. Neither study measured depressive symptoms, treatment beliefs, clinician recommendations and FLS participation within the same pathway. Administrative mental-disorder diagnoses may also capture differences in frailty, cognition, residence, comorbidity, continuity of care and competing clinical priorities. Antidepressant dispensing is particularly difficult to interpret because it may indicate current depression, past depression, anxiety, pain treatment or successful access to healthcare.

Depression should therefore be regarded as a potential stratification signal rather than an established explanation for disengagement. A recorded disorder or persistent symptoms may justify clarification of whether low mood, reduced motivation, impaired concentration or hopelessness is interfering with a defined next step. They do not justify coding treatment refusal or non-attendance as psychologically caused without evidence from the patient, caregiver or responsible clinician.

### 4.2 Concerns about falling: distinct from falls risk and most relevant to remobilisation

Concerns about falling are conceptually distinct from objectively assessed falls risk. Falls-risk assessment estimates the likelihood of a future fall using clinical history, physical performance, medication, cognition, vision and environmental factors. Concern about falling describes the person's appraisal of falling and its consequences, including balance confidence, falls-related self-efficacy and activity avoidance. The two constructs may coexist, but one cannot be inferred from the other.

This distinction has direct policy relevance. The existing FLS falls-assessment indicator can establish whether a falls assessment was completed or recommended, but it does not determine whether the patient is avoiding walking, exercise or rehabilitation because of concern about another fall. A patient may have substantial objective risk without marked concern, or marked activity-limiting concern despite relatively modest measured risk. These presentations require different responses.

A systematic review of 36 studies involving 5,099 participants found that reported fear-of-falling prevalence after hip fracture ranged from 22.5% to 100% (8). The range is not a credible single prevalence estimate; it reflects differences in terminology, instruments, thresholds and timing. The review found consistent associations between concerns about falling and measures of physical function, including balance, gait speed and self-reported function. It also found that only the Falls Efficacy Scale–International and Fear of Falling Questionnaire–Revised had adequate validation evidence in hip-fracture populations, while 10 of 14 intervention studies were judged at high risk of bias (8).

A mixed-methods review further showed that concerns about falling and activity restriction are produced by interacting physical, psychological, environmental, social and care-related factors (31). Balance, strength and mobility were relevant, but so were perceived control, confidence in rehabilitation, family support, access to clinicians, transport and accessibility of the home and outdoor environment. Consequently, an association between concern about falling and reduced activity should not automatically be interpreted as an isolated psychological mechanism.

One prospective study provides a more specific temporal signal. Falls-related self-efficacy assessed 6 weeks after hip-fracture surgery remained associated with 6-month functional outcomes after adjustment for age, pre-fracture function, cognition, pain and depressive symptoms (32). However, only 187 of 291 participants contributed 6-month outcomes, and patients lost to follow-up had greater baseline concern about falling. The result therefore supports further investigation of persistent concerns during rehabilitation but does not validate a universal 6-week screening threshold.

For policy purposes, concern about falling is most plausibly linked to rehabilitation acceptance, attendance, activity restriction and remobilisation rather than directly to osteoporosis-medication initiation. Assessment should focus on whether concern is limiting a specific activity or care step. Recording concern without identifying the affected behavior would produce a psychological label without an actionable service implication.

### 4.3 Engagement should be analyzed as a sequence of pathway nodes

Treatment engagement is not a single outcome. Within an FLS, it includes being reached by the service, attending assessment, understanding future fracture risk, participating in a treatment decision, initiating medication, commencing strength and balance intervention, completing early follow-up and sustaining treatment or self-management. A patient may complete one node and not the next. Combining these events under “adherence” obscures both the location and cause of pathway loss.

The transition from treatment recommendation to initiation illustrates this problem. In one study, 24.8% of patients did not start recommended osteoporosis pharmacotherapy. The most frequently reported reasons were fear of adverse effects, dislike of taking medicines and a belief that treatment would not help (33). Qualitative research similarly suggests that patients may understand the general concept of osteoporosis while remaining uncertain about the relationship between the fracture, future risk and the purpose of medication (34). These are not simply knowledge deficits. They involve interpretation of risk, anticipated harm, treatment burden and perceived necessity.

Mental-health and treatment-belief variables should therefore remain analytically separate. Depression may reduce motivation or capacity to act, while concern about adverse effects may represent a reasoned treatment preference. Concern about falling may inhibit rehabilitation but need not affect medication acceptance. Cognitive or communication difficulty may alter how a decision must be supported without invalidating the patient's preference. Practical barriers such as transport, cost, digital exclusion or unavailable follow-up may produce the same observable outcome as psychological disengagement.

Engagement is also co-produced by the service. FLS clinicians have reported difficulty communicating fracture risk and supporting shared decisions about osteoporosis medication (35). A missed appointment cannot be attributed to depression when accessible contact was not provided, and non-initiation cannot be attributed to patient reluctance when responsibility for prescribing was unclear. Reason attribution must therefore include patient-, clinician- and system-level explanations and allow more than one reason at each node.

The evidence identified in this review does not yet connect all elements of the proposed pathway. No prospective study identified in this review measured depression or concern about falling within an FLS population and then tested whether that measure independently predicted assessment attendance, treatment initiation, rehabilitation attendance or scheduled FLS follow-up. Evidence is strongest for general recovery after hip fracture and for associations between recorded mental disorders and treatment initiation in administrative cohorts. It is substantially weaker for the mechanisms producing attrition within an FLS.

This distinction defines the appropriate policy question. The issue is not whether depression and concerns about falling are associated with adverse outcomes in general. It is whether measuring and responding to them at a specified node improves completion of that node beyond what can be achieved through better communication, navigation and service organization. Table 3 maps these psychological and service-related explanations to specific FLS nodes, competing explanations, measurable outcomes and the strongest policy inference supported by the current evidence.

**Table 3:** Psychological and service factors mapped to FLS pathway nodes.

FLS node	Potential psychological contribution	Important competing explanations	Outcome that should be measured	Evidence status and permissible policy inference
Identification and first contact	Low motivation, distress or avoidance may reduce response	Incomplete case finding, inaccessible communication, incorrect contact details	Successful two–way contact	Author–derived inference. Psychological attribution requires patient, caregiver or clinical evidence
Assessment attendance	Depression or anxiety may make attendance difficult	Transport, pain, frailty, cognition, appointment design	Attendance or completed adapted assessment	Author–derived inference. Use reason attribution before selecting any screening target.
Treatment decision	Depressive cognition may affect information processing; medication concerns may shape preference	Poor risk communication, limited consultation time, clinical uncertainty	Decision quality and informed choice	Author–derived inference informed by indirect evidence. Declining treatment is not itself evidence of psychopathology
Medication initiation	Mood disorder may identify a group at risk of non–initiation	Prescribing responsibility, contraindication, cost, access and clinician inertia	Medication started within the specified period	Indirect evidence. The association supports targeted clarification, not causal attribution.
Rehabilitation initiation	Concern about falling may lead to activity avoidance	Pain, weakness, transport, programme availability	Attendance and commencement of rehabilitation	Indirect evidence. Confirm that concern is activity–limiting and potentially modifiable.
Early follow–up	Persistent symptoms may reduce response or continuity	Failed contact, transfer to primary care, fragmented records	Completed two–way follow–up	Author–derived inference. Record source–attributed reasons before assigning a cause.

This table is a conceptual mapping derived from the evidence synthesized in Sections 4.1–4.3 (7–10, 30–36). The listed psychological contributions are potential prognostic signals or explanatory hypotheses, not default causal assignments. Multiple patient, clinical and organizational explanations may coexist, and the evidence is drawn predominantly from hip–fracture populations rather than direct studies of FLS engagement. Concerns about falling are distinct from objectively assessed falls risk. The final column states the strongest policy inference considered permissible from the available evidence. FLS, fracture liaison service.

### 4.4 Intervention evidence does not yet establish an FLS engagement effect

The intervention literature must be interpreted according to the outcome an intervention was designed to change. Improvement in depressive symptoms is not evidence of improved medication initiation. Increased falls-related confidence is not evidence of increased physical activity. Better participation in decision-making is not necessarily evidence of medication persistence. These outcomes may each be valuable, but they should not be substituted for one another.

For depression, a systematic review of eight randomized trials involving 1,146 participants examined exercise-rehabilitation interventions after hip fracture (36). The pooled end-of-intervention effect on depressive symptoms favored rehabilitation but remained statistically imprecise, with a Hedges' g of −0.43 and a 95% confidence interval from −0.87 to 0.01. Interventions varied markedly in intensity, setting and additional components, and several adjusted analyses found no between-group difference. The review therefore suggested possible benefit without identifying a reliable FLS-deliverable intervention.

A more recent systematic review of 16 controlled studies involving 1,558 older adults reported improvement in psychosocial outcomes following theory-based psychological interventions but no statistically significant improvement in physical function (37). Risk of bias, heterogeneity and variable intervention content limited confidence in the result. Medication initiation, FLS attendance and follow-up completion were not established as intervention outcomes. The evidence consequently supports psychological benefit in selected hip-fracture populations but does not demonstrate that psychological treatment strengthens the secondary-fracture-prevention pathway.

Intervention evidence for concerns about falling reaches a similar conclusion. In the “Step by Step” randomized trial, a cognitive-behavioral intervention added to geriatric rehabilitation improved falls-related self-efficacy, with an effect size of −0.42 on the Short Falls Efficacy Scale–International (Short FES-I), but did not increase objectively measured daily walking duration (38). Other trials included in the systematic review were generally small or at high risk of bias, and exercise interventions with or without psychological components did not produce a consistent reduction in fear of falling (8). The divergence between confidence and walking behavior is especially important for FLS policy: a change in questionnaire score cannot be assumed to represent rehabilitation participation.

Evidence from an FLS-specific intervention points in the same direction. A quasi-experimental study combining a patient decision aid with motivational interviewing improved patients' reported involvement in osteoporosis-treatment decisions but did not significantly improve 1-year medication persistence compared with usual care−80.4% vs. 76.7%, respectively (39). The study was not randomized, but it provides a relevant warning against assuming that an improved consultation process will automatically change medication behavior.

Across these studies, additional clinician contact is a recurrent potential active ingredient. Psychological interventions often provide more time, follow-up and continuity than control care. Where benefit occurs, it may arise from navigation, attention or reinforcement rather than from a specific psychological technique. Patients with cognitive impairment, delirium, severe frailty or communication difficulty are also commonly excluded, despite being among those most likely to require adapted FLS engagement.

The evidence therefore supports three design requirements. First, an intervention must specify the psychological mechanism and the FLS node it is intended to protect. Second, evaluation must measure both the candidate psychological mechanism and the actual engagement outcome. Third, implementation outcomes—including reach, fidelity, workload, referral completion and equity—must be reported so that failure to deliver the intervention can be distinguished from failure of the intervention itself.

At present, the evidence supports targeted case finding and time-limited implementation studies where an actionable response exists. It does not support mandatory universal screening, routine psychological therapy delivered by all FLS teams, or incorporation of depression and concerns about falling into international performance standards. These alternatives are compared explicitly in the following section.

## 5 Assessment of policy options

### 5.1 Criteria for policy appraisal

Four policy options were assessed as progressively more intensive additions to existing FLS practice. They are not entirely mutually exclusive: reason attribution can precede targeted case finding, and both would be required before a universal pathway could be evaluated. The options were compared across six criteria.

First, policy fit asks whether the option addresses the identified problem—unexplained loss between FLS nodes—rather than psychological morbidity in general. Second, evidential proportionality considers whether the strength of the recommendation is commensurate with the predominantly observational and hip-fracture-based evidence. Third, actionability asks whether information generated by the option leads to a defined response. Fourth, feasibility includes staffing, training, information systems, referral capacity and opportunity cost to existing FLS functions. Fifth, autonomy and equity consider informed refusal, stigma and differential access for patients with cognitive, sensory, language or socioeconomic barriers. Sixth, evaluability asks whether reach, effectiveness, adoption, implementation and maintenance can be measured without conflating psychological, behavioral and clinical outcomes (40, 41).

These criteria deliberately place implementation burden and unintended effects alongside potential benefit. A policy that increases questionnaire completion but delays osteoporosis assessment, generates referrals that cannot be completed or excludes patients requiring communication support would not constitute successful integration. Table 4 compares the four policy options against these appraisal criteria and summarizes the resulting policy position.

**Table 4:** Comparison of policy options.

Policy option	Core content	Principal advantage	Main risk	Fit with evidence	Policy position
1. Status quo	Existing standards plus clinical discretion.	No additional burden; protects core work.	Inconsistent recognition and no explanatory learning.	Compatible but does not address the gap.	Temporary option where core FLS functions remain unstable.
2. Cross–node reason attribution	Structured, source–attributed reasons for non–completion.	Distinguishes patient, clinical and system explanations.	Misclassification, stigma and coding burden.	Strongest fit with current evidence and audit architecture.	Preferred immediate minimum.
3. Targeted case finding and response	Brief assessment after a trigger, linked to a named action.	Concentrates resources at an actionable node.	Missed cases, selection bias and unequal reach.	Supported as a testable extension.	Preferred where response capacity exists.
4. Universal stepped pathway	Routine assessment for all patients with graduated intervention.	Standardized reach and potential earlier identification.	Workload, medicalisation, unmet need and displacement of core care.	Evidence insufficient for routine adoption.	Multicentre research option only.

The four options are analytically distinct but may be implemented sequentially; they are not assumed to be mutually exclusive over time. Appraisal was qualitative and used the criteria specified in Section 5.1: evidence directness and consistency, expected benefit, implementation burden, response capacity, patient autonomy, equity, opportunity cost and preservation of core FLS performance. This comparison is not a formal GRADE assessment, health–technology assessment or cost–effectiveness analysis. The policy positions are the authors' recommendations based on the critical synthesis. FLS, fracture liaison service; GRADE, Grading of Recommendations Assessment, Development and Evaluation.

### 5.2 Option 1: retain current standards and rely on clinical discretion

Under the status quo, FLS teams would continue to deliver existing identification, assessment, treatment, falls and follow-up functions. Depression or concerns about falling could be addressed when recognized clinically, but no additional reason taxonomy, psychological case finding process or response indicator would be required.

The principal advantage is preservation of capacity. Many services have not yet achieved reliable case identification, timely assessment, treatment initiation or long-term monitoring (15, 18). Requiring additional data or clinical work in such settings may divert staff time from interventions with stronger evidence. Clinical discretion also permits experienced practitioners to respond flexibly without turning distress after fracture into a mandatory screening outcome.

The limitations are inconsistency and low explanatory value. Recognition depends on individual staff awareness, consultation time and patient disclosure. Services cannot determine whether psychological barriers contribute systematically to non-completion, compare pathway loss across groups or evaluate whether an informal response was provided. Patients whose difficulty is not immediately visible may receive no adapted support, while similar presentations may be handled differently across services.

The status quo may be defensible temporarily where the core FLS pathway is absent, under-resourced or performing poorly. It is insufficient as the preferred long-term policy for established services because it leaves the identified attribution problem unchanged and provides no basis for learning whether a psychologically informed response would improve engagement.

### 5.3 Option 2: introduce cross-node reason attribution without routine screening

The second option adds a structured reason field whenever an eligible FLS step is not completed. It does not require a depression or concern-about-falling questionnaire. Instead, the service records the best available explanation for non-completion, permits multiple contributing reasons, identifies the source of the information and retains “unknown” as a valid response.

A minimum taxonomy would distinguish failure to establish contact, informed refusal, unresolved treatment uncertainty, clinical ineligibility or deferral, cognitive or communication support needs, a patient-reported psychological barrier, practical access problems, transfer of responsibility and service failure. Attribution would occur at the relevant node rather than being applied retrospectively to the entire pathway.

This option directly addresses the principal policy gap identified in Section 3. It can distinguish potentially modifiable service problems from informed patient decisions without presuming that a psychological disorder is present. It is also compatible with existing FLS audit logic because it adds explanatory information to established process outcomes rather than creating a parallel clinical programme. Aggregated reason data could show, for example, whether poor initiation primarily follows unresolved medication concerns, failure of prescribing transfer or inability to contact patients.

The data will nevertheless be vulnerable to misclassification. Clinicians may infer depression from non-attendance, record “patient declined” when discussion was incomplete or select a psychological category when the barrier was inaccessible care. Reason attribution could also become a mechanism for transferring responsibility from the service to the patient. These risks require operational definitions, staff training, source attribution, multiple-reason coding and periodic comparison of coded reasons with patient or caregiver accounts.

Option 2 represents the most proportionate immediate policy change. It is relatively low burden, generates information needed to design later interventions and can identify whether a service's dominant problems are psychological, organizational, practical or shared. It should initially be treated as a quality-improvement measure rather than a national performance target. Services should be assessed on credible completion and use of the data, not on achieving a low rate of refusal or psychological barriers.

### 5.4 Option 3: targeted case finding linked to a named response

The third option adds brief psychological case finding when a predefined trigger indicates that depression or concern about falling may threaten a specific FLS node. Potential triggers include a documented mental disorder, patient- or caregiver-reported persistent low mood, repeated failure to complete an agreed care step, unresolved treatment uncertainty, or concern about falling that restricts rehabilitation or everyday activity.

Case finding should follow a two-stage model. An initial question would establish whether the factor is present and relevant to the next care step. A validated instrument could then be used when greater clarification would change management. The objective would not be to diagnose every mental disorder. It would be to determine whether a potentially modifiable factor is interfering with assessment, treatment initiation, rehabilitation or follow-up.

Every measure would require a named response and responsible actor. Responses within routine FLS competence could include adapted communication, teach-back, clarification of medication concerns, involvement of a caregiver with consent, proactive follow-up or coordination with rehabilitation. Persistent, severe or clinically complex findings would be referred to an existing primary-care, geriatric, rehabilitation or mental-health pathway. Referral generation alone would not count as completion; the service would need a method for confirming acceptance or establishing an alternative plan.

This option has a stronger evidence-to-action relationship than universal screening. It concentrates resources where the proposed factor is already linked to an observable engagement problem and accommodates the evidence that depression and concerns about falling are unlikely to operate identically across all nodes. Depression-related difficulty may be most relevant to treatment initiation or follow-up, whereas activity-limiting concern about falling is more directly relevant to remobilisation and rehabilitation.

Targeting also creates risks. Patients who do not disclose symptoms or whose barriers are not recognized may be missed. Staff judgements may reproduce inequities, particularly for people with cognitive impairment, limited health literacy, language barriers or atypical presentations. A trigger based only on missed appointments would identify difficulty after it had already affected care. Trigger criteria, reach and missingness should therefore be examined across patient groups rather than assuming that targeted delivery is inherently equitable.

Option 3 is the preferred clinical extension where four conditions are met: the service can define the threatened node; a brief and valid measure is available for the population; a response can be delivered or coordinated; and completion can be reassessed. In the absence of these conditions, targeted case finding risks producing information without care.

### 5.5 Option 4: a universal stepped mental-health-informed FLS pathway

The most intensive option would offer standardized assessment of depressive symptoms and concerns about falling to all eligible patients, followed by progressively more intensive responses according to need. Assessment might occur at the initial FLS encounter and again during early follow-up, recognizing that acute distress may resolve while persistent difficulty may become more apparent during rehabilitation.

A universal pathway offers several theoretical advantages. It reduces dependence on clinician recognition, produces standardized denominators, may identify patients who would not otherwise disclose difficulty and permits comparison across services. When embedded within a stepped response, it could align fracture prevention with wider person-centered care for older people (26).

The current evidence does not establish that these benefits would outweigh the burden. The psychological literature is dominated by hip-fracture populations, and no identified trial demonstrated that universal assessment within an FLS improved assessment attendance, treatment initiation, rehabilitation participation or follow-up. Available interventions improved some symptom, confidence or decision-process outcomes without consistently changing objective behavior (37–39). Universal implementation would therefore extend beyond the populations, settings and outcomes in which the evidence was generated.

Screening would also create clinical responsibility. Positive findings require review, clarification, safety assessment where indicated, referral capacity and follow-up. Without these components, screening may increase documentation without improving care. It may identify understandable and transient distress as pathology, increase anxiety, create waiting lists or expose needs that the service cannot address. Repeated measurement could be particularly burdensome for patients experiencing pain, fatigue, cognitive impairment or multiple appointments.

Opportunity cost is central. FLS programmes operate with variable staffing and incomplete achievement of existing quality indicators (18). Time spent administering and processing psychological measures may delay fracture-risk assessment, medication recommendation or patient follow-up. Universal screening could consequently improve the visibility of mental-health need while weakening the fracture-prevention functions the service was established to provide.

Option 4 should therefore be treated as an implementation hypothesis rather than a current standard. It may be appropriate for multicentre feasibility or hybrid effectiveness–implementation studies in services with stable core performance and established response pathways. It should not yet be incorporated into international FLS accreditation or national audit targets.

### 5.6 Preferred policy sequence

The preferred policy is a staged sequence rather than immediate universal integration.

The first stage is cross-node reason attribution. Established FLS programmes should improve their ability to explain why eligible patients do not complete major pathway steps, while retaining informed refusal, multiple causation and unknown reasons.

The second stage is targeted case finding. Services with credible response capacity should assess depression or concerns about falling when there is a defined clinical or engagement trigger and when the result can change an action. The psychological measure, target node, responsible actor and reassessment point should be specified in advance.

The third stage is multicentre evaluation. Universal or broader stepped pathways should be tested only after reason categories, targeted measures and response processes have demonstrated feasibility. Evaluation should examine patient reach, equity, staff adoption, fidelity, cost, sustainability and preservation of existing FLS performance, as well as the engagement outcome the intervention is intended to change (40, 41).

Progression between stages should not be automatic. A service should pause or redesign the model if positive findings do not receive a documented response, if referral capacity is inadequate, if participation is systematically lower among disadvantaged groups or if timely bone-health assessment and treatment deteriorate. Conversely, a targeted pathway should be considered for wider testing if it reaches the intended population, is acceptable to patients and staff, improves the prespecified engagement node and does not materially weaken core secondary prevention.

This sequence does not position mental-health integration as an alternative to effective FLS delivery. It applies the same quality-improvement logic on which FLSs are based: identify where preventable loss occurs, assign responsibility, introduce a proportionate response and measure whether care reaches the patient.

## 6 Actionable recommendations

The recommendations operationalise cross-node reason attribution as the immediate minimum policy and targeted case finding as a conditional extension. They do not recommend universal psychological screening as a current FLS standard. Implementation should begin with a defined engagement problem, use existing contacts wherever possible and proceed only when the service can respond safely to the information collected.

### 6.1 Establish a minimum attribution dataset before adding screening

Current evidence supports piloting a structured reason field at major non-completed transitions, initially covering assessment attendance, treatment recommendation, medication initiation, strength and balance intervention, early monitoring and longer-term treatment status. The minimum record should identify the node, eligibility, completion status, one or more contributing reasons, attribution source, response provided, responsible professional and reassessment outcome. ‘Unknown' must remain a valid category; requiring certainty where none exists would improve apparent completeness while reducing validity.

The reason taxonomy should use the operational definitions in Supplementary Table S4. Informed refusal requires an accessible discussion, an opportunity for questions and a documented decision; unsuccessful contact requires failure of two-way contact after the locally prespecified number and modes of attempts; clinical deferral requires a named clinical reason and review plan; cognitive or communication need requires the support need and adaptation offered; practical access barriers include transport, cost, caregiving, timing or digital exclusion; and service failure includes absent ownership, an unissued prescription or referral, lost results, unavailable services or failed hand-off. Patient-reported psychological barrier is reserved for low mood, anxiety or activity-limiting concern about falling reported or clinically assessed as affecting the selected node. Multiple secondary codes are permitted, and “unknown” is preferable to unsupported attribution.

### 6.2 Use targeted case finding only when a named response is available

#### 6.2.1 Select the engagement node before selecting a measure

Each pilot should identify one priority node from existing data, such as non-attendance at assessment, failure to initiate recommended medication, non-commencement of strength and balance intervention or failure to complete early follow-up. It should then state the proposed mechanism. Persistent low mood may reduce treatment initiation; activity-limiting concern about falling may reduce rehabilitation attendance; cognitive or communication difficulty may prevent effective follow-up. Baseline performance should be established using a supplementary all-eligible transition denominator where the official KPI is conditioned on a previous recommendation.

#### 6.2.2 Apply brief, two-stage case finding

Case finding should occur only at a prespecified decision point and when acute delirium, unstable illness or an inability to participate does not make the result uninterpretable: at the first successful FLS assessment when a relevant concern is reported; immediately after a selected node is not completed; at the next routine rehabilitation or follow-up contact, commonly 4–6 weeks after fracture or discharge, when difficulty persists; or at the 12–16-week follow-up when an initial positive finding or incomplete node requires reassessment. Triggers are a recorded mental disorder, patient- or caregiver-reported persistent low mood, repeated difficulty completing an agreed step, unresolved treatment concern, or concern about falling that restricts a named activity or rehabilitation step. For depressive symptoms, the proposed low-burden sequence is PHQ-2 followed by PHQ-9 when the PHQ-2 score is at least 3 or clinical concern persists (11, 12). For concerns about falling, two gateway questions establish concern and activity restriction. Once relevant activities can be judged, the FES-I has the stronger validation basis after hip fracture; the Short FES-I is a lower-burden alternative when a locally validated language version is used and the result will change the rehabilitation response (8, 13). Scores indicate symptoms or concern, not diagnosis or causal attribution, and no questionnaire threshold alone should determine why an FLS node was not completed. Any response to the PHQ-9 self-harm item requires immediate clarification and activation of the organization's established safety pathway.

#### 6.2.3 Deliver a four-step response within existing contacts

Step 1-recognize and clarify: before psychological attribution, check pain intensity and activity interference, acute illness and adverse effects, delirium or cognitive change under the local clinical protocol, communication and sensory access, treatment understanding and preference, transport and cost, appointment notification, prescription or referral status, and service availability. If delirium, acute cognitive change or unstable illness prevents valid self-report, defer questionnaire-based case finding, address the clinical issue and reassess at the next appropriate contact. The source of each explanation must remain visible. More than one explanation may be recorded, and the primary reason should be the factor judged most proximal to the selected node after source review. Immediate safety concerns should activate the organization's established urgent pathway rather than a separate FLS emergency system.

Step 2—respond within routine FLS competence: use node-specific communication and support, such as clarification of fracture risk or a medication concern, teach-back, shared decision-making, one achievable rehabilitation step, adapted information, caregiver involvement with consent or proactive scheduling. Generic reassurance or another leaflet is insufficient when the barrier is unresolved ambivalence, low motivation or activity avoidance.

Step 3—coordinate additional support: when need exceeds FLS competence, coordinate with primary care, rehabilitation, falls services, pain management, geriatric medicine, liaison psychiatry, clinical psychology or older-adult mental-health services. Referral generation is not completion; a closed loop requires confirmation of acceptance, patient or caregiver understanding and explicit interim responsibility.

Step 4—reassess the target node: record both the proposed barrier and the behavior it was intended to influence. If the step remains incomplete, reassess the reason rather than carrying it forward automatically. Psychological state, preference, clinical circumstances and service access may change over time.

#### 6.2.4 Assign responsibility before expanding case finding

FLS leaders and commissioners should map which findings remain within routine FLS care, which require external input, the responsible professional, expected response times, how information returns to the FLS and who retains responsibility while the patient waits. Staff require competence in recognizing node-relevant difficulty, avoiding unsupported causal attribution, communicating risk, supporting informed decisions and activating local escalation procedures; they do not need to become mental-health diagnosticians.

Figure 2 translates these recommendations into a minimum viable targeted-response pathway, from a node-specific trigger through attributed reason, named response and reassessment of the original care step.

**Figure 2:**
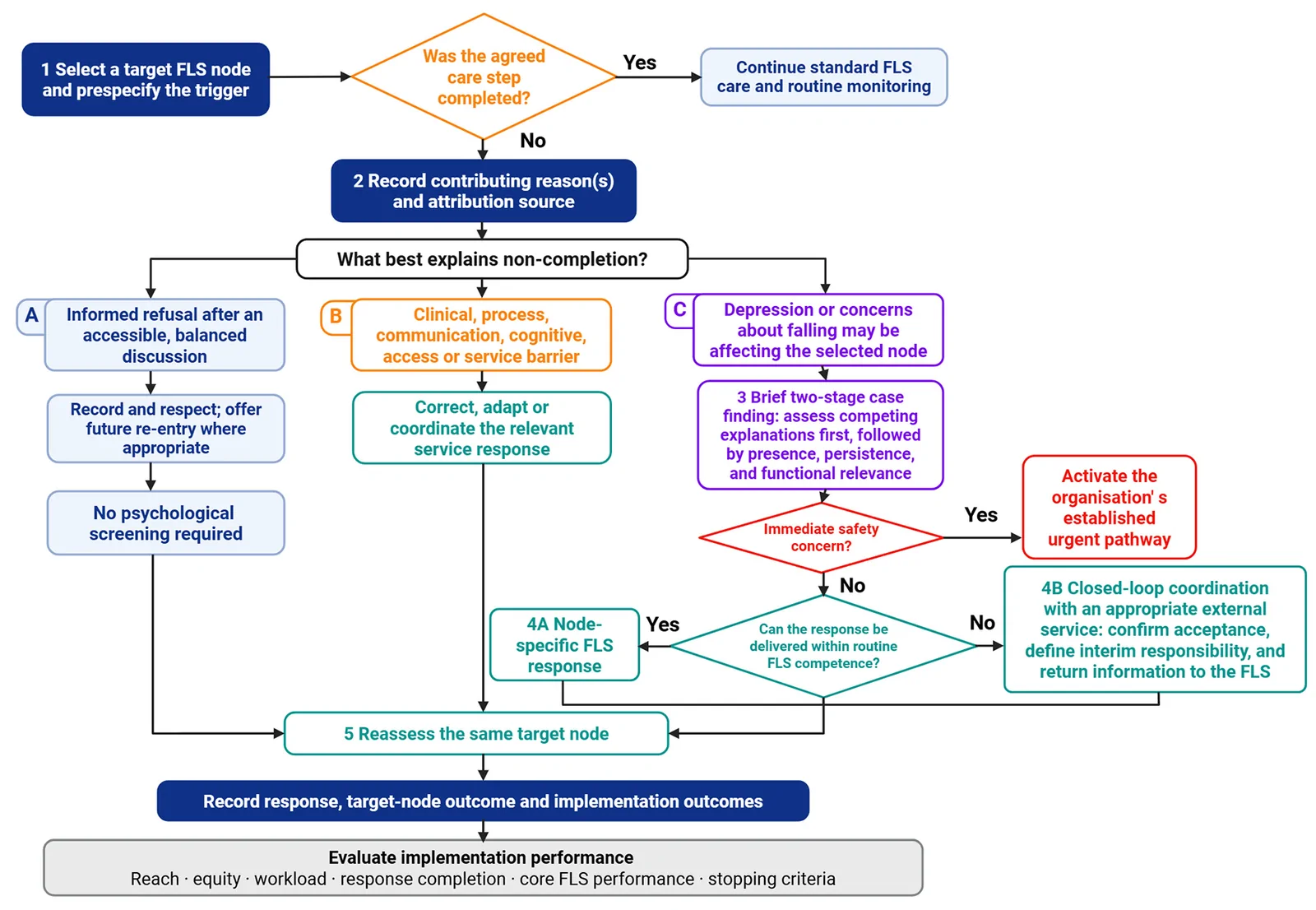
Minimum viable response pathway for targeted mental-health-informed FLS care. A prespecified, node-specific trigger initiates source-attributed assessment of the reason or reasons for non-completion. Informed refusal is recorded and respected; remediable clinical, process, communication, access or service barriers prompt correction within existing care; possible depression or concerns about falling prompt brief, two-stage case finding only where a named response is available; and immediate safety concerns activate the organization's established urgent-risk pathway. The original care node is then reassessed. Color key: navy denotes the core FLS workflow and required documentation; pale blue denotes standard-care continuation and the informed-refusal branch; orange denotes attribution decisions and remediable non-psychological barriers; purple denotes conditional mental-health case finding; red denotes urgent safety escalation; teal denotes service response, closed-loop coordination and reassessment; gray denotes implementation evaluation and stopping rules; and black arrows and Yes/No labels denote direction and neutral decision logic. Colors distinguish functional roles in the proposed pathway and do not represent an evidence hierarchy. Case finding is not universal and should be introduced only where response capacity exists. FLS, fracture liaison service BioRender.com.

### 6.3 Protect autonomy, equity and clinical safety

A patient who declines medication after a balanced and comprehensible discussion has completed a legitimate decision-making process. Psychological attribution should require patient or caregiver report or a documented clinical assessment, and the source should remain visible. A missed appointment should not be attributed to depression when contact was inaccessible, interpretation unavailable or the appointment not communicated successfully. The purpose of attribution is to locate a remediable problem, not assign blame.

Patients requiring caregiver involvement, translated information, hearing or visual adaptation, home-based contact or non-digital follow-up should remain within the eligible denominator. Services should report reach, data completeness, response and target-node completion across locally relevant equity variables. Missing psychological data should not be treated as random: inability to complete a questionnaire may itself indicate mismatch between pathway and need. Embedding the response in existing contacts should be the default to avoid creating an additional, inequitable attrition point.

### 6.4 Evaluate a time-limited multicentre pilot before setting standards

Before implementation, each site should prespecify the target population and node, baseline performance, triggers, measure and threshold, response and responsible actor, expected workload and referral demand, equity strata, progression criteria and stopping conditions. Evaluation should distinguish implementation outcomes from psychological, behavioral and clinical outcomes. Reach, acceptability and fidelity establish whether the pathway was delivered; change in symptoms or confidence tests the proposed mechanism; completion of the selected node tests whether the behavior changed; and existing KPIs test whether the added work displaced core care (40, 41). Table 5 specifies the proposed implementation indicators, denominators and safeguards for a time-limited multicentre pilot.

**Table 5:** Proposed implementation indicators for a pilot.

Indicator	Proposed numerator and denominator	Interpretation and safeguard
Reason attribution	Non–completed eligible steps with a source–attributed reason/all non–completed eligible steps	Measures explanatory completeness, not patient compliance
Pathway reach	Eligible patients completing the minimum assessment/all eligible patients	Report reasons for missingness and relevant equity strata
Positive–finding response	Positive findings receiving the predefined response/all positive findings	A positive result without action represents incomplete implementation
Closed–loop coordination	Referrals accepted, attended or replaced by a documented alternative/all referrals generated	Do not count referral generation alone
Target–node completion	Patients completing the prespecified FLS node/all eligible for that node	Preserve informed refusal as a legitimate outcome
Psychological outcome	Patients with a clinically or meaningfully improved measure/patients receiving the targeted response	Do not use this as a proxy for behavioral change
Core–service preservation	Change in existing FLS KPIs, waiting time and caseload during implementation	Added activity should not materially displace core fracture prevention
Equity	Reach, response and node completion across relevant patient groups	Investigate differential missingness and exclusion
Acceptability and burden	Patient and staff acceptability, completion time and discontinuation	Interpret alongside workload and qualitative explanations
Sustainability	Continued delivery after initial implementation support/services beginning the pilot	Wider adoption requires a sustainable workforce and funding model

These are proposed measures for a time-limited multicentre implementation pilot and are not validated KPIs or national standards. Before use, each service should prespecify the assessment window, data source, responsible actor, relevant equity strata, target threshold and stopping or modification rule. Denominators should retain informed refusal and relevant missingness unless a justified exclusion is reported. Improvement in a psychological measure should not be used as a proxy for completion of the targeted FLS node. Implementation domains are informed by Proctor and RE-AIM (40, 41). FLS, fracture liaison service; KPI, key performance indicator; RE-AIM, Reach, Effectiveness, Adoption, Implementation and Maintenance.

A service should pause or redesign the pathway if psychological assessment delays bone-health assessment or treatment, positive findings repeatedly receive no response, referral waiting times make the promised pathway unavailable, or reach is systematically lower among patients requiring cognitive, communication or socioeconomic support. Progression requires acceptable reach and fidelity, a functioning response pathway, preservation of core FLS performance and a signal of improvement in the prespecified engagement outcome. Symptom improvement alone should not justify scale-up when the target behavior remains unchanged.

IOF, national audit programmes and professional organizations should not yet add universal depression or concern-about-falling screening as a mandatory performance indicator. They could support harmonization and testing of a cross-node reason taxonomy, source attribution, minimum implementation outcomes and node-specific engagement outcomes. A psychologically informed indicator should be considered for benchmarking only after multicentre evidence demonstrates feasibility, interpretability, equitable reach and benefit without deterioration in existing FLS performance.

## 7 Discussion

### 7.1 Principal findings and policy contribution

This review identifies a policy problem at the intersection of FLS quality measurement and post-fracture psychological care. International FLS frameworks provide a strong architecture for identification, assessment, treatment recommendation and follow-up, but their audit logic more readily records whether selected steps occurred than consistently links non-completion to an attributed reason, its source, a service response and the subsequent outcome (14, 15, 18). This distinction matters because informed refusal, unresolved uncertainty, psychological difficulty, communication need and service failure require different interpretations and responses.

The psychological evidence provides plausible signals rather than direct explanations of FLS pathway loss. Depression has been associated with poorer recovery and lower treatment initiation in post-fracture cohorts (7, 9, 10, 30). Concerns about falling have been associated with activity restriction and functional outcomes, particularly during remobilisation and rehabilitation (8, 31). These studies were largely conducted outside FLS settings and do not isolate psychological explanations from pain, frailty, cognition, treatment beliefs, environmental constraints or service organization. Intervention studies identified in this review did not use completion of a prespecified FLS engagement node as the primary outcome (36–39). The policy implication is therefore limited to testing node-specific hypotheses, not assigning psychological causes by default.

The review's contribution is operational: it distinguishes the observed non-completed transition, the reported reason and its source, the clinician's interpretation, the response delivered and subsequent completion. This structure supports prospective testing but should not be interpreted as validation of the proposed taxonomy.

### 7.2 Relationship to current FLS policy and practice

The proposed approach extends the quality-improvement logic of the IOF framework rather than altering the core FLS model. Existing architecture makes key processes and responsible services visible (14, 15). Adding explanatory information at selected non-completed transitions could apply the same logic to pathway loss while retaining current indicators as measures of core delivery.

Implementation would necessarily be context-sensitive. Asia-Pacific consensus and review evidence emphasize adaptation, education, staffing and coordination (19, 20); Canadian studies identify organizational readiness and change management (21, 22); Australian consensus work highlights unresolved responsibility for longer-term monitoring (23); and Chinese benchmarking shows that strong inpatient performance may coexist with post-discharge loss (24). Taken together, these sources support treating mental-health-informed FLS care as an organizational intervention whose actors, referral routes and follow-up arrangements must be specified locally, rather than as a questionnaire transferred unchanged across systems.

The England and Wales FLS-DB is informative as a worked case, not a global template. It demonstrates that a mature audit can measure pathway stages at scale and record selected states such as decline or non-contactability (15, 18). The absence of a consistent cross-node linkage between reason, attribution source, response and subsequent outcome does not imply that individual services provide no psychosocial care; it identifies a limitation of what can be reconstructed from routine audit data. The proposed extension is correspondingly bounded: the FLS would identify whether a possible barrier affects its own next care step, respond within competence or coordinate an accepted referral, retain interim responsibility as locally agreed and reassess the target node. These functions require prospective testing before any audit or accreditation requirement is considered.

### 7.3 Why universal screening is not yet justified

Universal screening is not supported by the current evidence. It would create duties for interpretation, response, documentation, referral and urgent escalation in services with variable staffing and referral capacity. The evidence is concentrated in selected hip-fracture and rehabilitation populations, with limited representation of people with cognitive impairment, delirium, severe frailty or communication difficulty. The proportionate alternative is targeted enquiry after source-attributed clarification of the affected node and only where a response is available. Research measurement across a cohort should not be treated as a universal clinical screening requirement. Broader use should be considered only after multicentre evaluation demonstrates added value, equitable reach, acceptable burden and preservation of core FLS performance.

### 7.4 Autonomy, equity and unintended effects

Autonomy is not an ancillary safeguard. It determines what counts as successful engagement. A patient who declines treatment after an accessible, balanced discussion has completed a legitimate decision-making process. Similarly, avoiding activity may reflect pain, environmental hazard, inadequate support or a realistic appraisal of risk, rather than a psychological barrier. Labeling either outcome as disengagement would create measurement error and could turn quality improvement into pressure to reach a clinician-preferred decision.

Attribution must also accommodate co-produced and changing explanations. Patient, caregiver and clinician accounts, and administrative records, provide different kinds of information; their source should remain visible, multiple reasons should be permitted and disagreement should be recorded rather than forced into a single presumed truth. A reason identified at one contact should be reassessed because mood, preferences, clinical circumstances and service access may change. This approach separates explanation from blame and makes divergence between accounts informative for service redesign.

Equity is both an ethical and a validity issue. Questionnaire completion and follow-up may be lower among people with cognitive impairment, limited literacy, sensory loss, language barriers or digital exclusion. Removing these patients from denominators can make an intervention appear feasible by selectively excluding those who need adaptation. Evaluation should therefore report who was reached, what adaptation or proxy input was used, how missingness differed between groups and whether the intended response and target-node outcome were achieved. Proxy reports should be identified as such rather than treated as interchangeable with the patient's account.

### 7.5 Evidence required before policy adoption

Research should now determine whether the proposed attribution-and-response pathway adds useful explanation, changes a defined FLS transition and preserves existing care sufficiently to justify policy adoption. Further documentation that psychological distress occurs after fracture would not answer that policy question.

Prospective multicentre cohorts should examine depression and concerns about falling separately, at prespecified times, against node-specific outcomes. A minimum covariate set covering pain, frailty, cognition, treatment beliefs, social support and service exposure would test added explanatory value without imposing a universal measurement battery. Outcomes should distinguish successful contact, assessment attendance, informed decision or refusal, medication initiation, participation in an indicated strength-and-balance or rehabilitation intervention, early follow-up and longer-term treatment status. Research measurement in this design would not itself constitute a recommendation for routine screening.

The proposed reason taxonomy requires prospective mixed-method validation before policy adoption. Content validity and comprehensibility should be co-developed with patients, caregivers, FLS clinicians, rehabilitation staff, primary care, mental-health professionals and audit leads, with explicit review of stigma and equity. Similar healthcare classification studies have used explicit coding rules, independent coding before adjudication and complementary agreement measures to identify ambiguous or infrequently assigned categories. At each site, the reliability sample should include consecutive or randomly selected non-completed transitions and be justified by the expected category prevalence and the desired precision of the agreement estimates. Two trained coders should classify the same source-labeled records independently before adjudication, using an explicit manual and worked examples. Patient, caregiver, clinician and administrative accounts must remain separate, and disagreement between sources must not be treated as coder error. Reliability reporting should include category prevalence, a primary-category confusion matrix, percent agreement and Cohen's kappa with 95% confidence intervals, plus category-specific positive and negative agreement for multi-label secondary codes. The codebook, training and decision rules should be revised and the sample recoded when prespecified progression criteria are not met. High inter-rater reliability would establish reproducibility of coding, not construct validity, causal truth or clinical utility. Feasibility pilots should then test attribution, targeted case finding, response and same-node reassessment as a coherent process, reporting reach, missingness, staff time, acceptability, fidelity, completed safety escalation, referral closure, opportunity cost and unintended effects (40, 41). Psychological measures should be treated as candidate prognostic markers or hypothesized mechanisms; claims of effect modification or mediation would require prespecified statistical, temporal and causal assumptions.

Comparative effectiveness should follow only after response capacity and delivery feasibility are demonstrated. The design should match the intervention level and risk of contamination; cluster-randomized or stepped-wedge approaches are options rather than methodological defaults. The primary outcome should be completion of the targeted node, with symptoms, confidence, quality of life and implementation outcomes analyzed separately. Early economic work should use a stated perspective and time horizon to examine costs and consequences, including effects on existing FLS activity; full economic evaluation requires an appropriate comparator and credible effectiveness estimate. A core outcome set should be co-developed with patients, caregivers, FLS staff and policy stakeholders. Poor reach, unclosed referrals, unequal delivery, unchanged target-node outcomes or deterioration in core performance would argue against progression; consistent benefit without material displacement would support wider testing rather than immediate standardization.

### 7.6 Strengths and limitations

The principal strength of this review is that it connects international FLS quality architecture, post-fracture psychological evidence, treatment engagement and implementation science around a single policy question. Its node-based framework was designed to reduce unsupported movement from an association to a service recommendation and to preserve distinctions among patient preference, psychological difficulty and organizational failure. The England and Wales documentary case adds operational detail while remaining explicitly bounded as one national audit example.

The review also has important methodological limitations. It is a critical narrative synthesis, not a systematic review: structured database searching was limited to PubMed; selection and extraction were not performed independently by two reviewers; the initial yield of targeted policy searches and citation chasing was not enumerated; and no *de novo* study-level risk-of-bias assessment was undertaken. The selection flow was reconstructed from preserved search outputs and reference ledgers after peer review, and contemporaneous item-level reasons for all title/abstract exclusions were unavailable. Policy and audit findings are version-dependent. These constraints limit completeness and reproducibility despite the added search log, PMID set, exclusion log and evidence map.

External and construct validity are further constrained by the evidence itself. Most psychological studies concern hip-fracture populations, often exclude people with cognitive or communication needs and use heterogeneous definitions of depression and concern about falling. Most evidence is observational and vulnerable to confounding. Intervention studies generally measure symptoms, confidence or function rather than FLS node completion. The proposed taxonomy and response pathway are unvalidated; attributed reasons may change over time, and disagreement between sources may reflect genuine differences in perspective rather than recording error.

Accordingly, the review supports a bounded sequence for prospective evaluation, not an effectiveness claim. The recommendations identify what could be piloted, what must be measured and what evidence would be required before broader standards are considered.

## 8 Conclusion

Fracture liaison services have made secondary fracture prevention organized, accountable and measurable. Their current architecture identifies whether core care steps are delivered but does not consistently explain why successive transitions are not completed. Depression and concerns about falling are plausible contributors to this loss, but the evidence remains predominantly observational, centered on hip fracture and insufficient to establish causation or mandate universal screening.

The proportionate policy response is staged. Services should first make non-completion attributable, distinguishing informed refusal, psychological difficulty and service failure. Targeted case finding should follow only where a defined FLS node is at risk and a named response is available. Universal stepped pathways should be evaluated in multicentre studies before consideration as national or international standards.

Mental-health integration should therefore be treated as a testable FLS quality-improvement strategy. Its success should be judged not by the number of questionnaires completed, but by whether the intervention reaches patients equitably, produces an appropriate response, improves the targeted engagement node and preserves the core work of secondary fracture prevention.
