# Supplementary material for: Integrating mental health into fracture liaison services: an attribution-first policy approach to depression, concerns about falling and pathway loss
Source: Front Public Health. 2026 Oct 26;14:1989494. doi: 10.3389/fpubh.2026.1989494 (PMC13745348; doi:10.3389/fpubh.2026.1989494)
Supplement: Supplementary file 1 [file Table_1.docx]

Supplementary Material

This supplement reports the search strategies, source-selection account, full-text exclusions, evidence map, proposed attribution codebook and targeted case-finding specification used in the revised review.

**Supplementary Table S1A. Bibliographic database searches**

Database note. The four PubMed strategies were updated and rerun on 31 August 2026. Counts are raw returns at the time of searching. Records were deduplicated by PMID across the four searches; no language or study-design filter was applied at the search stage.

| **Database / source** | **Search date** | **Coverage** | **Search domain** | **Search strategy** | **Records retrieved** | **Notes** |
| --- | --- | --- | --- | --- | --- | --- |
| PubMed | 31 Aug 2026 | Database inception–31 Aug 2026; no language or study-design filter at search stage | FLS policy, standards, audit and quality | (("fracture liaison service"[Title/Abstract] OR "fracture liaison services"[Title/Abstract]) AND (policy[Title/Abstract] OR policies[Title/Abstract] OR guideline*[Title/Abstract] OR standard*[Title/Abstract] OR audit*[Title/Abstract] OR benchmark*[Title/Abstract] OR "quality improvement"[Title/Abstract] OR "quality indicator"[Title/Abstract] OR "quality indicators"[Title/Abstract] OR "performance indicator"[Title/Abstract] OR "performance indicators"[Title/Abstract])) | 240 | Raw yield before de-duplication and screening; official NCBI E-utilities execution. |
| PubMed | 31 Aug 2026 | Database inception–31 Aug 2026; no language or study-design filter at search stage | FLS implementation and engagement | (("fracture liaison service"[Title/Abstract] OR "fracture liaison services"[Title/Abstract]) AND (implement*[Title/Abstract] OR feasib*[Title/Abstract] OR acceptab*[Title/Abstract] OR fidelity[Title/Abstract] OR sustainab*[Title/Abstract] OR adoption[Title/Abstract] OR "organizational readiness"[Title/Abstract] OR "organisational readiness"[Title/Abstract] OR "change management"[Title/Abstract] OR engagement[Title/Abstract] OR attendance[Title/Abstract] OR follow-up[Title/Abstract] OR persistence[Title/Abstract] OR adherence[Title/Abstract] OR refusal[Title/Abstract] OR nonattendance[Title/Abstract])) | 397 | Raw yield before de-duplication and screening; official NCBI E-utilities execution. |
| PubMed | 31 Aug 2026 | Database inception–31 Aug 2026; no language or study-design filter at search stage | Depression or mental disorder and post-fracture osteoporosis treatment | ((fracture*[Title/Abstract] OR "hip fracture"[MeSH Terms]) AND (depress*[Title/Abstract] OR "mental disorder"[Title/Abstract] OR "mental disorders"[Title/Abstract] OR psychiatric[Title/Abstract] OR antidepressant*[Title/Abstract]) AND (osteoporosis[Title/Abstract] OR "Osteoporosis"[MeSH Terms]) AND ("treatment initiation"[Title/Abstract] OR initiation[Title/Abstract] OR uptake[Title/Abstract] OR adheren*[Title/Abstract] OR persist*[Title/Abstract] OR medication[Title/Abstract] OR pharmacotherap*[Title/Abstract])) | 121 | Raw yield before de-duplication and screening; official NCBI E-utilities execution. |
| PubMed | 31 Aug 2026 | Database inception–31 Aug 2026; no language or study-design filter at search stage | Concerns about falling and post-fracture rehabilitation or participation | (("hip fracture"[Title/Abstract] OR "hip fractures"[Title/Abstract] OR "fragility fracture"[Title/Abstract] OR "fragility fractures"[Title/Abstract] OR "osteoporotic fracture"[Title/Abstract] OR "osteoporotic fractures"[Title/Abstract]) AND ("fear of falling"[Title/Abstract] OR "concern about falling"[Title/Abstract] OR "concerns about falling"[Title/Abstract] OR "falls efficacy"[Title/Abstract] OR "fall-related self-efficacy"[Title/Abstract] OR "balance confidence"[Title/Abstract]) AND (rehabilitat*[Title/Abstract] OR mobility[Title/Abstract] OR "physical activity"[Title/Abstract] OR "activity restriction"[Title/Abstract] OR participation[Title/Abstract] OR recovery[Title/Abstract] OR function*[Title/Abstract])) | 152 | Raw yield before de-duplication and screening; official NCBI E-utilities execution. |
| PubMed total | 31 Aug 2026 | As above | Four domains combined | Union of the four searches above | 910 across searches; 746 unique PMIDs | 746 is the de-duplicated database set, not the number of included records. |

**Supplementary Table S1B. Targeted policy, guideline, standards and audit searches**

Targeted-source note. Counts were not assigned to website searches because changing site architecture and duplicate versions make raw web-result counts non-reproducible and analytically uninformative. Instead, the table reports the search pathway and the exact authoritative records retained. Superseded audit reports may be used only for temporal comparison; the 2026 dataset and calculation documents should support current claims.

| **Database / source** | **Search date** | **Coverage** | **Search domains / approach** | **Official records retained for policy analysis** | **Notes** |
| --- | --- | --- | --- | --- | --- |
| International Osteoporosis Foundation (IOF) / Capture the Fracture | 31 Aug 2026 | Official Capture the Fracture resource centre, Best Practice Framework, guidelines/policies and benchmarking/audit pages | Site navigation plus targeted terms: "Best Practice Framework", "fracture liaison service", KPI, benchmark, audit, policy, guideline; backward citation checking of framework documents. | 1. [Best Practice Framework for Secondary Fracture Prevention (2017 operational framework)](https://www.capturethefracture.org/sites/default/files/pdf-bpf-framework/2017-IOF-CTF-best_practice_framework-EN-WEB.pdf)  2. [Åkesson et al. Capture the Fracture: a Best Practice Framework and global campaign (2013)](https://doi.org/10.1007/s00198-013-2348-z)  3. [Javaid et al. Patient-level FLS key performance indicator set (2020)](https://doi.org/10.1007/s00198-020-05377-1)  4. [Capture the Fracture Best Practice Framework web resource](https://www.capturethefracture.org/best-practice-framework)  5. [Capture the Fracture benchmarking and audit resource](https://www.capturethefracture.org/index.php/resource-center/you-already-have-a-pfc/benchmarking-and-audit) | Primary international architecture for FLS process standards and benchmarking; used to establish what current frameworks measure and what they do not specify. |
| World Health Organization (WHO) | 31 Aug 2026 | WHO publications portal; healthy ageing, integrated care and person-centred assessment resources | Targeted terms: "integrated care for older people", ICOPE, psychological capacity, mobility, social support, person-centred pathways. | 1. [Integrated care for older people (ICOPE): guidance for person-centred assessment and pathways in primary care, 2nd ed. (2025)](https://www.who.int/publications/i/item/integrated-care-for-older-people-%28-icope%29-guidance-for-person-centred-assessment-and-pathways-in-primary-care) | Contextual integrated-care framework; not an FLS-specific standard and should not be cited as proof that mental-health screening improves FLS engagement. |
| National Osteoporosis Guideline Group (NOGG) | 31 Aug 2026 | NOGG 2024 guideline website and downloadable full guideline; updated December 2024 | Site navigation plus targeted terms: FLS, fracture liaison, coordinator, audit, database, follow-up, adherence, falls. | 1. [NOGG 2024 Clinical guideline for the prevention and treatment of osteoporosis](https://www.nogg.org.uk/full-guideline)  2. [Section 9: Models of care for fracture prevention](https://www.nogg.org.uk/full-guideline/section-9-models-care-fracture-prevention)  3. [Section 11: Recommendations for commissioners of healthcare](https://www.nogg.org.uk/full-guideline/section-11-recommendations-commissioners-healthcare) | Current UK osteoporosis and FLS care-model guidance; supports coordinator-based services and local audit, but does not establish a universal FLS mental-health pathway. |
| National Institute for Health and Care Excellence (NICE) | 31 Aug 2026 | Current NICE guidance and quality standards relevant to falls, fragility fracture, osteoporosis risk assessment and hip-fracture management | Targeted terms: falls, concerns about falling, mood, cognition, fragility fracture, osteoporosis, hip fracture, rehabilitation, quality standard. | 1. [NG249: Falls—assessment and prevention (2025)](https://www.nice.org.uk/guidance/ng249)  2. [CG146: Osteoporosis—assessing the risk of fragility fracture (2012; updated 2017)](https://www.nice.org.uk/guidance/cg146)  3. [QS149: Osteoporosis quality standard (2017)](https://www.nice.org.uk/guidance/qs149)  4. [CG124: Hip fracture—management (2011; updated 2023, amended 2025)](https://www.nice.org.uk/guidance/cg124) | NG249 is the principal UK guideline source for mood, cognition and concerns about falling within multifactorial falls assessment; other records define fracture and osteoporosis care context. |
| Royal College of Physicians / Falls and Fragility Fracture Audit Programme (RCP/FFFAP) | 31 Aug 2026 | FLS-DB official website, current audit downloads, public benchmark tables, resource repository and annual reports | Site navigation plus targeted terms: FLS-DB, dataset, data dictionary, KPI calculations, missed opportunities, benchmark, non-attendance, declined, treatment, follow-up, adherence. | 1. [FLS-DB annual report 2026: Steps to fracture liaison service effectiveness](https://www.rcp.ac.uk/improving-care/national-clinical-audits/falls-and-fragility-fracture-audit-programme-fffap/fracture-liaison-service-database-fls-db/fls-db-annual-report-2026/)  2. [FLS-DB general audit downloads](https://www.fffap.org.uk/fls/flsweb.nsf/docs/Downloads)  3. [V6 Patient dataset proforma (2026)](https://www.fffap.org.uk/fls/flsweb.nsf/docs/Downloads)  4. [Current KPI calculations](https://www.fffap.org.uk/fls/flsweb.nsf/docs/Downloads)  5. [Current missed-opportunities calculations](https://www.fffap.org.uk/fls/flsweb.nsf/docs/Downloads)  6. [FLS-DB public benchmarks and run charts](https://www.fffap.org.uk/FLS/charts.nsf/benchmarks?ReadForm=&org1=&vw=BALL&yr=2025)  7. [FLS-DB resource repository and service-improvement materials](https://www.rcp.ac.uk/resources/fls-db-resource-repository/) | Worked UK audit case. The dataset, denominator specifications and calculation documents—not the annual report alone—must be used to verify claims about reason attribution and conditional denominators. |
| Royal Osteoporosis Society (ROS) | 31 Aug 2026 | ROS clinical publications and FLS implementation resources | Targeted terms: "clinical standards for Fracture Liaison Services", implementation toolkit, service specification, patient pathway, KPI. | 1. [Clinical standards for Fracture Liaison Services (August 2019)](https://theros.org.uk/media/1eubz33w/ros-clinical-standards-for-fracture-liaison-services-august-2019.pdf)  2. [FLS implementation toolkit](https://theros.org.uk/for-healthcare-professionals/clinical-quality-hub/clinical-quality-toolkits/fracture-liaison-services/fls-implementation-toolkit/) | UK service-standard and implementation context; cited by FLS-DB as part of the basis for its KPI set. |
| Backward citation checking | 31 Aug 2026 | Reference lists of included systematic/scoping reviews, consensus statements and foundational FLS framework papers | Backward checking for additional policy, implementation and empirical sources relevant to a defined FLS node or service response. | Additional records were retained only when they informed a prespecified policy question and their bibliographic details and claim use could be verified. | Citation checking supplemented, but did not convert, the review into a PRISMA-governed systematic review. |

**Supplementary Figure S1. Reconstructed source-selection flow**

Counts were reconstructed from preserved PubMed search outputs, reference ledgers and source-verification records. The initial record-level yield from targeted policy, guideline and audit websites and backward citation checking was not preserved. Title and abstract exclusion reasons were not retained at the individual-record level. This figure documents the reconstructed selection account for this critical narrative Policy and Practice Review and should not be interpreted as a prospective PRISMA flow diagram. Screening was not conducted independently in duplicate.

**Supplementary Table S2A. Reconstructed selection accounting**

| **Stage** | **Count** |
| --- | --- |
| PubMed records across four searches | 910 |
| Cross-search duplicates removed | 164 |
| Unique PubMed records screened | 746 |
| Title/abstract records not retained | 726 |
| PubMed full-text reports assessed | 20 |
| Other-route full-text reports assessed before revision | 21 |
| Post-review measurement validation reports assessed | 3 |
| Total full-text reports assessed | 44 |
| Full-text reports excluded | 3 |
| Sources included in revised synthesis | 41 |

**Supplementary Table S2B. Full-text exclusions**

Reasons are report-specific and correspond to the three full-text exclusions in Supplementary Figure S1.

| **Report** | **PMID** | **Reason for exclusion** |
| --- | --- | --- |
| Peairs et al. The association of pre-existing mental health conditions and patient outcomes after lower extremity orthopaedic trauma: a scoping review. | 37955721 | Wrong population for the principal policy question: the review excluded studies with mean age above 65 years, limiting applicability to older FLS populations. |
| Smith et al. What factors affect early mobilisation following hip fracture surgery: a scoping review. | 38253357 | Wrong scope/outcome: focused on early postoperative mobilisation rather than depression, concerns about falling, osteoporosis-treatment engagement or an FLS node. |
| Alvarado et al. Adherence to anti-osteoporotic therapy and five-year survival after fragility hip fracture. | 41701251 | Five-year survivor-only adherence outcome; the report did not test psychological attribution or a targeted FLS engagement mechanism. |

**Supplementary Table S3A. Brief evidence map by primary domain**

Each of the 41 included sources was assigned one mutually exclusive primary domain. Source-level assignments are supplied in Supplementary Dataset S1.

| **Primary evidence domain** | **n** | **Predominant directness** | **Permissible use** | **Claim boundary** |
| --- | --- | --- | --- | --- |
| FLS model, effectiveness and implementation | 14 | Predominantly direct FLS evidence | FLS processes, outcomes, audit gaps and implementation experience | Does not establish that a mental-health intervention improves FLS completion |
| Policy, guideline, audit and integrated care | 7 | Direct FLS or contextual evidence | Current standards, service architecture and policy gaps | Supports policy context, not intervention effectiveness |
| Depression or mental disorder after fracture | 5 | Predominantly indirect evidence | Post-fracture prevalence, associations and clinical relevance | Does not establish FLS-specific causality or attribution |
| Concerns about falling and rehabilitation | 6 | Predominantly indirect evidence | Relationship with activity restriction, rehabilitation and recovery | Does not prove that concern caused non-completion of an FLS node |
| Treatment beliefs, non-initiation and shared decisions | 4 | Indirect evidence | Patient-reported reasons, perceived need and decision-making | Context-specific; cannot be assumed for an individual patient |
| Implementation and evaluation frameworks | 2 | Contextual evidence | Framework for feasibility and implementation evaluation | Does not demonstrate clinical effectiveness |
| Measurement validation | 3 | Indirect measurement evidence | Measurement properties of PHQ-2, PHQ-9 and FES-I/Short FES-I | A positive score is not a diagnosis or causal attribution |

**Supplementary Table S3B. Evidence-directness categories**

| **Category** | **Sources** | **Operational meaning** |
| --- | --- | --- |
| Direct FLS evidence | 17 | Generated within an FLS or by an FLS-specific framework or audit and addresses an identifiable pathway process. |
| Indirect evidence | 14 | Generated in post-fracture or osteoporosis populations outside a defined FLS; supports plausibility or measurement, not an FLS-specific causal claim. |
| Contextual evidence | 10 | Adjacent clinical guidance, audit, policy, implementation or measurement framework used to define context and options. |
| Author-derived inference | Not counted as source evidence | Proposed taxonomy, causal hypotheses, policy-option appraisal and staged implementation sequence; explicitly unvalidated. |

**Supplementary Table S4A. Proposed cross-node reason-attribution codebook**

The taxonomy is author-developed and intended for prospective validation. Multiple secondary codes are allowed and unknown or mixed remains a valid code.

| **Code** | **Operational definition** | **Permitted attribution source** | **Safeguard** |
| --- | --- | --- | --- |
| Unsuccessful contact | No two-way contact after the locally prespecified number and modes of attempts. | Administrative contact log | Do not infer refusal or psychological cause. |
| Informed refusal | Patient declines after accessible information, opportunity for questions and documented decision-making. | Patient; caregiver only where authorised | Record as a legitimate outcome, not pathway failure. |
| Unresolved uncertainty or treatment concern | Patient reports unanswered concern, low perceived need, burden or ambivalence that prevents the node. | Patient or caregiver report | Separate from depression and from informed refusal. |
| Clinical ineligibility or deferral | Acute illness, contraindication, prognosis, pain or another clinical reason delays or precludes the node. | Clinician assessment plus record | Name the clinical reason and planned review date. |
| Cognitive or communication support need | Delirium, cognitive impairment, sensory/language barrier or inaccessible information affects participation. | Patient/caregiver/clinician; tool if used | Record adaptation offered and whether it enabled completion. |
| Patient-reported psychological barrier | Low mood, anxiety or activity-limiting concern about falling is reported as affecting the named node. | Patient/caregiver report or documented clinical assessment | A scale score alone does not prove causation. |
| Practical or social access barrier | Transport, cost, caregiving, digital exclusion, timing or home circumstances impede the node. | Patient/caregiver/admin record | Keep distinct from motivation or refusal. |
| Transfer or service failure | Unclear ownership, unissued prescription/referral, lost result, unavailable service or failed hand-off. | Administrative/clinical record | Allocate organisational owner and corrective action. |
| Unknown or mixed | Available information is insufficient or several reasons remain plausible. | All sources retained separately | Unknown is valid; allow multiple secondary codes. |

**Supplementary Table S4B. Proposed reliability evaluation**

| **Reliability field** | **Specification** |
| --- | --- |
| Pilot sample | Two independent coders classify at least 50 consecutive non-completed transitions per site or 10%, whichever is larger. |
| Blinding | Code from the same source-attributed record before adjudication; do not merge patient, caregiver, clinician and administrative accounts. |
| Statistics | Report category prevalence, a primary-category confusion matrix, percent agreement and Cohen's kappa with 95% confidence intervals, plus category-specific positive and negative agreement for multi-label secondary codes. |
| Progression | Prespecify acceptable reliability and retrain/revise the codebook before effectiveness testing; do not infer validity from high agreement alone. |

**Supplementary Table S5. Targeted case-finding specification**

Assessment is triggered by a named node problem and used only when an actionable response is available; it is not universal screening. Scores indicate symptoms or concern, not diagnosis or causal attribution.

| **Timing** | **Trigger criteria** | **Preferred tools** | **Competing explanations to check first** | **Required action** |
| --- | --- | --- | --- | --- |
| First successful FLS contact or assessment | Known mental disorder; patient/caregiver reports persistent low mood; activity-limiting concern about falling; communication/cognitive difficulty | PHQ-2 for depressive symptoms; two gateway questions on concern and activity restriction; when relevant activities can be judged, FES-I has the stronger post-hip-fracture validation basis; Short FES-I is a lower-burden alternative using a locally validated language version. | Pain intensity/interference; delirium or cognitive status under local protocol; communication access; acute illness | Clarify relation to the selected node and name an immediate response. |
| Immediately after a prespecified node is not completed | Non-attendance, non-initiation, non-commencement of rehabilitation or failed follow-up | Reason-attribution interview first; PHQ-2 to PHQ-9 if PHQ-2 is 3 or greater or clinical concern persists; FES-I for persistent activity-limiting concern after hip fracture, or Short FES-I as a locally validated lower-burden alternative. | Appointment notification, transport, cost, prescription/referral status, adverse effects, pain, cognition and patient preference | Do not administer a psychological measure until service and clinical explanations are checked. |
| Next routine rehabilitation or follow-up contact, usually 4–6 weeks after fracture/discharge | Persistent or new difficulty relevant to rehabilitation or treatment | Repeat the same tool only if monitoring will alter support; use a locally validated language version. | Recovery stage, analgesia, delirium resolution, new complications, programme availability | Document response completion and reassess the same FLS node. |
| Early follow-up, usually 12–16 weeks or the service-defined KPI contact | Initial positive finding or prior incomplete node | PHQ-9 or the same baseline concern-about-falling measure only when clinically appropriate and interpretable. | Medication access, rehabilitation exposure, cognition, social support and service continuity | Measure symptoms separately from target-node completion; stop or redesign if no response capacity. |

**Supplementary Dataset S1. Search and evidence dataset**

The accompanying Supplementary Dataset S1 provides the four PubMed search strategies, reconstructed selection counts, report-specific full-text exclusions and the source-level evidence map.

Abbreviations: CI, confidence interval; FES-I, Falls Efficacy Scale-International; FFFAP, Falls and Fragility Fracture Audit Programme; FLS, fracture liaison service; FLS-DB, Fracture Liaison Service Database; IOF, International Osteoporosis Foundation; KPI, key performance indicator; NICE, National Institute for Health and Care Excellence; NOGG, National Osteoporosis Guideline Group; PHQ-2, two-item Patient Health Questionnaire; PHQ-9, nine-item Patient Health Questionnaire; PMID, PubMed identifier; PRISMA, Preferred Reporting Items for Systematic Reviews and Meta-Analyses; RCP, Royal College of Physicians; ROS, Royal Osteoporosis Society; WHO, World Health Organization.
